# Relationship Between Medical Questionnaire and Influenza Rapid Test Positivity: Subjective Pretest Probability, “I Think I Have Influenza,” Contributes to the Positivity Rate

**DOI:** 10.7759/cureus.16679

**Published:** 2021-07-28

**Authors:** Masahito Katsuki, Mitsuhiro Matsuo

**Affiliations:** 1 Department of Neurosurgery, Itoigawa General Hospital, Itoigawa, JPN; 2 Department of Internal Medicine, Itoigawa General Hospital, Itoigawa, JPN; 3 Department of Anesthesiology, Graduate School of Medicine and Pharmaceutical Sciences, University of Toyama, Toyama, JPN

**Keywords:** automated artificial intelligence (autoai), influenza, medical interview, subjective pretest probability, rapid influenza diagnosis test, deep learning, prediction one

## Abstract

Introduction

Rapid influenza diagnostic tests (RIDTs) are considered essential for determining when to start influenza treatment using anti-influenza drugs, but their accuracy is about 70%. Under the COVID-19 pandemic, we hope to refrain from performing unnecessary RIDTs considering droplet infection of COVID-19 and influenza. We re-examined the medical questionnaire’s importance and its relationship to the positivity of RIDTs. Then we built a positivity prediction model for RIDTs using automated artificial intelligence (AI).

Methods

We retrospectively investigated 96 patients who underwent RIDTs at the outpatient department from December 2019 to March 2020. We used a questionnaire sheet with 24 items before conducting RIDTs. The factors associated with the positivity of RIDTs were statistically analyzed. We then used an automated AI framework to produce the positivity prediction model using the 24 items, sex, and age, with five-fold cross-validation.

Results

Of the 47 women and 49 men (median age was 39 years), 56 patients were RIDT positive with influenza A. The AI-based model using 26 variables had an area under the curve (AUC) of 0.980. The stronger variables are subjective pretest probability, which is a numerically described score ranging from 0% to 100% of “I think I have influenza,” cough, past hours after the onset, muscle pain, and maximum body temperature in order.

Conclusion

We easily built the RIDT positivity prediction model using automated AI. Its AUC was satisfactory, and it suggested the importance of a detailed medical interview. Both the univariate analysis and AI-based model suggested that subjective pretest probability, “I think I have influenza,” might be useful.

## Introduction

In Japan, rapid influenza diagnostic tests (RIDTs) are considered essential tools for determining when to start the treatment for influenza using anti-influenza drugs, in addition to behavioral measures like hand washing and using masks. This is because the Japanese people want to know the results of RIDTs rather than a qualitative clinical diagnosis by a doctor, although the diagnostic accuracy of RIDTs is around 70-80% [1]. Also, in Japan, patients need the certification of the RIDT results, regardless that they are positive or negative, to go to work or be on sick leave when they have colds during the influenza pandemic season. Therefore, Japanese clinicians routinely use RIDTs as a complementary exam with influenza-like illnesses, and most positive patients are treated with anti-influenza drugs [2]. Doctors also tend to depend on RIDTs because there are few specific physical findings to influenza [3]. However, the treatment for influenza is sometimes initiated based on clinical diagnosis despite negative RIDTs. Therefore, unnecessary RIDTs are being conducted, putting pressure on medical costs. Furthermore, under the COVID-19 pandemic, RIDTs should be performed for patients with a high pretest probability of influenza considering droplet infection of both COVID-19 and influenza.

To reduce unnecessary RIDTs and prepare for the telemedicine era under the COVID-19 pandemic [4], we herein re-examined the medical interview’s importance and its relationship to the positivity of RIDTs. Then we preliminarily built a prediction model for the positive rate of RIDTs using detail medical interview results and one of the automated artificial intelligence (AI) frameworks, Prediction One (Sony Network Communications Inc., Tokyo, Japan) [5-11].

## Materials and methods

Study population

We retrospectively retrieved data from medical records of all the consecutive 96 patients who underwent RIDTs at the outpatient department from December 2019 to March 2020. We used a questionnaire sheet before conducting RIDTs, and doctors performed RIDTs using ESPLINE Influenza A and B-N® (Fujirebio, Tokyo, Japan). We collected the sample at the epipharynx using a swab through the nasal cavity.

Items in the questionnaire sheet

The questionnaire sheet consisted of the following 24 items: the time duration from the onset, maximum body temperature, vaccination status, pretest probability numerically described as a subjective probability of having influenza ranging from 0% to 100%, and contact with a sick person. We also asked about the presence of detailed symptoms on a three-point scale: heat sensation, tiredness, chills, cold sweat, muscle pain, muscle pain, joint pain, headache, sneezing, shoulder ache, runny nose, nasal congestion, sore throat, cough, sputum, stomach ache, nausea, diarrhea, lower back pain, and feeling “kowai,” a local word describing vague physical ailments (Table 1).

**Table 1:** Medical questionnaire before rapid influenza diagnostic tests *a local word describing vague physical ailments

Onset	( ) hours ago			
Maximum body temperature	( )℃			
Vaccination	Done	Not yet		
Pretest probability	You think “I have influenza as ( )% of probability.”			
Sick contact	Family	School	Workplace	Other ( )
	( ) days ago			
	Never			
Please check whether you have symptoms of any as following	Yes	Neither	No	
Heat sensation	+1	0	-1	
Tiredness	+1	0	-1	
Chills	+1	0	-1	
Cold sweat	+1	0	-1	
Muscle pain	+1	0	-1	
Joint pain	+1	0	-1	
Headache	+1	0	-1	
Sneezing	+1	0	-1	
Shoulder ache	+1	0	-1	
Runny nose	+1	0	-1	
Nasal congestion	+1	0	-1	
Sore throat	+1	0	-1	
Cough	+1	0	-1	
Sputum	+1	0	-1	
Stomach ache	+1	0	-1	
Nausea	+1	0	-1	
Diarrhea	+1	0	-1	
Lower back pain	+1	0	-1	
“Kowai”*	+1	0	-1	

Statistical analysis

Results were derived as median (interquartile range). The difference between patients with RIDT positive and those negative was examined using univariate analysis with logistic regression. A two-tailed p < 0.05 was considered statistically significant. We calculated p-values and odds ratios (OR) using EZR, which is a graphical user interface for R (The R Foundation for Statistical Computing, Vienna, Austria) [12], and SPSS Statistics software version 21.0.0. (IBM, Armonk, New York, USA). For numerical variables, the adjusted odds ratios were computed by the exponentiation of the coefficient calculated by the logistic regression. We did not perform multivariate analysis due to the small sample size and many variables, so we built a prediction model using the automated AI framework, Prediction One, instead [5].

Building a prediction model by Prediction One

We used Prediction One to build the prediction model. We used all 96 patients' data with 26 variables, including the 24 items described above plus sex and age. The objective variable is positive or negative in the RIDTs. Prediction One read the 96 patients’ data and automatically divided them into five-fold cross-validation datasets. Prediction One automatically adjusted and optimized the variables that are easy to be processed statistically and mathematically and select appropriate algorithms with ensemble learning. The missing values were automatically compensated, and Prediction One built the best prediction model by an artificial neural network with internal five-fold cross-validation. The details of functions are trade secrets and could not be provided. The area under the curve (AUC) of the model and strong variables with the weights in the artificial neural network were automatically calculated. The model’s AUC, accuracy, precision, recall, and F-value were used to evaluate the prediction model built by AI.

Ethics

Our hospital’s research ethics committee approved this study (ethical approval number 2019-20). We gained written informed consent for this study from all of the patients, the legally authorized representative of the patients, or next of kin. All methods were carried out under relevant guidelines and regulations (Declaration of Helsinki).

## Results

Clinical characteristics

The clinical characteristics of the 96 patients (47 women and 49 men) are summarized in Table 2. The median (interquartile range) age was 39 (26-55). Fifty-six patients (58%) were positive in RIDTs. All the patients had the influenza A virus. The univariate analysis revealed that muscle pain (OR 1.60, 95% confident interval [CI] 1.01-2.56), cough (OR 3.62, 95%CI 1.80-9.26), and the pretest probability, the score numerically described as a subjective probability of having influenza ranging from 0% to 100% (OR 1.02, 95%CI 1.01-1.04), were related to the positivity rate in RIDTs. The results of the univariate analysis are shown in Table 2.

**Table 2:** Characteristics of the patients Results are shown as median (interquartile range). RIDTs: rapid influenza diagnostic tests, *a local word describing vague physical ailments, **p-value < 0.05, ^†^logistic regression calculated odds ratio and 95% confident interval

Variables	RIDTs Positive (n = 56)	RIDTs Negative (n = 40)	Odds ratio (95% confident interval)†
Age (years)	40 (26-51)	29 (22-45)	1.09 (0.99-1.01)
Women: Men	25:31	22:18	0.97 (0.42-2.32)
Onset-to-presentation interval, < 6 h	8	6	0.92 (0.29-2.89)
Maximum body temperature	38.5 (38.3-39.0)	38.5 (37.6-39.1)	1.20 (0.72-1.99)
Vaccination	32	16	1.92 (0.8-4.34)
Pretest probability	60 (50-80)	40 (10-50)	1.02 (1.01-1.04)**
Sick contact	29	16	1.61 (0.71-3.63)
Detailed questionnaire items (Yes: Neither: No)			
Heat sensation	51:1:4	34:2:4	1.31 (0.65-2.65)
Tiredness	49:5:2	31:3:6	1.83 (0.90-3.72)
Chills	43:3:10	25:6:9	1.41 (0.84-2.36)
Cold sweat	29:11:16	7:8:25	1.38 (0.84-2.27)
Muscle pain	31:9:16	13:10:17	1.60 (1.01-2.56)**
Joint pain	36:6:14	19:4:17	1.51 (0.96-2.36)
Headache	33:8:15	24:4:12	1.03 (0.65-1.63)
Sneezing	19:9:28	5:12:23	1.53 (0.92-2.54)
Shoulder ache	24:6:26	12:8:20	1.22 (0.78-1.90)
Runny nose	34:8:14	16:8:16	1.58 (0.99-2.50)
Nasal congestion	15:7:34	8:8:24	1.09 (0.67-1.77)
Sore throat	33:6:17	23:5:12	1.01 (0.65-1.59)
Cough	48:7:1	22:5:13	3.62 (1.80-7.26)**
Sputum	28:8:20	14:6:20	1.41 (0.91-2.21)
Stomach ache	3:11:42	2:8:30	1.01 (0.49-2.09)
Nausea	7:12:37	6:6:28	1.03 (0.59-1.81)
Diarrhea	2:7:47	4:7:29	0.58 (0.28-1.19)
Lower back pain	29:6:21	17:6:17	1.18 (0.76-1.82)
“Kowai”*	28:9:19	22:9:19	0.83 (0.51-1.36)

AI model development

Prediction One produced an AI-based prediction model using the 96 patients with 26 variables in less than 40 seconds. The AUC of the model was 0.980. The model’s accuracy, precision, recall, and F-value were 0.916, 0.887, 0.982, and 0.932, respectively. Its sensitivity and specificity were 0.982 and 0.825, respectively.

The stronger variables and their weights of the model are listed in Table 3. Pretest probability, presence of cough, past hours after the onset, presence of muscle pain, maximum body temperature, presence of sputum, feeling “kowai,” age, presence of joint pain, and cold sweat are important factors, in that order.

**Table 3:** Stronger variables in the RIDT positivity prediction model built by automated AI AI: artificial intelligence, RIDTs: rapid influenza diagnostic tests, *the score numerically described as a subjective probability of having influenza ranging from 0% to 100%, ^†^a local word describing vague physical ailments

Order of strength	Variables	Weight
1	Pretest probability*	0.127
2	Cough	0.111
3	Onset-to-presentation interval, < 6 h	0.099
4	Muscle pain	0.086
5	Maximum body temperature	0.084
6	Sputum	0.080
7	“Kowai”^†^	0.079
8	Age	0.073
9	Joint pain	0.069
10	Cold sweat	0.061

## Discussion

We administered a carefully designed medical questionnaire to the patients with influenza-like symptoms. We found that muscle pain, cough, and pretest probability, a numerically described subjective score ranging from 0% to 100% of “I think I have influenza,” were related to the positive rate in RIDTs in the univariate analyses. Also, we built result prediction model for RIDTs using Prediction One from the 96-patient dataset with the detailed medical questionnaire. Our results offer two suggestions: 1) pretest probability was related to the positivity of RIDTs, and 2) this is the first report on an AI-based prediction model using a detailed medical questionnaire for the positivity rate of RIDTs.

Clinical diagnosis for influenza

Clinical diagnosis is essential before laboratory tests to increase pretest probability. It is ideal to perform RIDTs on people with suspected influenza based on clinical diagnosis. However, in Japan, RIDTs are uniformly performed on patients with influenza-like symptoms during influenza epidemics without a careful medical interview. This is because there are so many outpatients with cold symptoms during winter that we cannot provide usual medical services with careful medical interviews. Also, patients need "certification" based on the RIDTs' results to be allowed to go to work or be on sick leave, so too many patients come to the outpatient department. Therefore, too many meaningless RIDTs are performed in Japan, and it is desired to reduce them to save the limited medical resources.

In a guideline on the diagnosis and management of influenza [13], it was suggested selected individuals should be tested for influenza if the results are anticipated to influence management decisions or a public health response. Therefore, acute febrile respiratory illnesses in patients who are not at high risk for influenza complications and who do not require hospital admission can sometimes be diagnosed as influenza with a high likelihood by clinical criteria alone [14]. In a retrospective analysis of signs and symptoms in 3,744 ambulatory patients with an influenza-like illness who participated in phase II and III trials of neuraminidase inhibitors during outbreaks, the best predictor was the combination of fever and cough within 48 hours of the development of symptoms, which had a positive predictive value of 79% for influenza [1]. Boivin et al.’s study on children suggested that cough and fever were the only clinical factors associated with positive RT-PCR (reverse transcription polymerase chain reaction) results [15]. Call et al.’s study revealed that a combination of fever, cough, and acute onset is related to influenza with a 2.0 positive likelihood ratio [16]. These studies have shown that clinical symptoms help diagnose influenza. However, there are no reports on the usefulness of the subjective pretest probability of having influenza ranging from 0% to 100%. Our report is the first report on its utility.

AI and influenza diagnosis

Previous studies on influenza diagnosis using AI remain few. Xia et al. reported an AI-based classifier distinguishing influenza from COVID-19 using chest X-ray images and clinical features with an AUC of 0.9 [17]. Choo et al. reported the usefulness of the patient-generated health data obtained from a mobile health application to develop an AI-based screening tool for influenza [18]. However, Japan has lagged behind other countries in introducing such mobile health applications based on smartphones or smartwatches [19]. Matsui et al. developed a non-contact influenza screening system within 30 s using the newly developed testers for vital signs [20]. Dagdanpurev et al. then developed an AI-based screening model for influenza using those patients’ vital signs [21]. Lopez et al. studied AI-based influenza detection systems using natural language processing technology and concluded that a free-text record would help detect influenza [22]. Although AI-based influenza screening systems are being developed, they are still few. Our study is similar to the previous reports on the attitudes to reduce unnecessary RIDTs and predict RIDTs' test results with AI technology. We suggested a possibility that a detailed medical interview could predict RIDTs' results depending on AI technology without contact with patients. In the future, an automatically diagnosing system using Japanese natural language processing [8,23] for the free text in the medical questionnaire could be invented as a developmental form of our model.

Advantages of AI

Conventional time- and cost-consuming statistical analysis need the arbitrary selection of variables based on previous studies, and multivariate analysis needs 10-15 folds number of samples against the variables [24]. Therefore, there is a risk that variables that might be important may not be included in the statistical analysis. Even the multivariate analysis cannot be performed in a small hospital with a small dataset like ours. However, automated AI like Prediction One develops beneficial models with less effort or time using the small dataset, without time-consuming variable optimization or arbitrarily choosing variables. Also, the number of variables used in the framework is not limited, and AI sometimes finds interesting variables that have not been taken into account in the previously reported statistical models as important [25].

We then review these benefits in our study. Conventionally, we could have used only nine variables for the statistical analysis due to the small sample size of the dataset (n = 96). However, we could use 26 variables for building the prediction model by Prediction One, and also build a good prediction model from the small dataset. Furthermore, we could not perform multivariate analysis due to the small sample size, but Prediction One suggested important variables as if AI considered confounders. Besides, the time needed for creating each model was less than 40 seconds. Finally, the models achieved high accuracy with an AUC of 0.980. We could not perform validation due to the small sample size, but our study suggested the possibility of making the RIDT positivity prediction model only from the detailed medical questionnaire. This technology would help us refrain from performing unnecessary RIDTs considering the medical costs and the COVID-19 pandemic. It also spurs the developing telemedicine era.

Limitation of this study

First, the sample size was so small that we did not perform validation. We only detected important variables using univariate analysis and automated AI. The model’s performance is unknown for validation. Second, it is uncertain that the prediction model derived from our own data can be applied to other institutions and those using other RIDTs.

## Conclusions

We easily and quickly built the RIDT positivity prediction model using Prediction One. Its AUC is satisfactory, and it suggested to us the importance of a detailed medical interview. Especially, subjective pretest probability “I think I have influenza” would be useful. Although validation process is needed, our AI-based prediction model suggested the potential that influenza could be diagnosed with a detailed medical interview and that our preliminary results might be used for further AI-related research like telemedicine and automated diagnosis using natural language processing.
